# Hybrid imaging with PET/CT and PET/MR

**DOI:** 10.1186/1470-7330-14-S1-O32

**Published:** 2014-10-09

**Authors:** Andreas Kjaer

**Affiliations:** 1 Dept. of Clinical Physiology, Nuclear Medicine & PET, Rigshospitalet, University of Copenhagen, 2000 Copenhagen, Denmark

## 

Hybrid PET/MRI systems have recently become commercially available with potential to change medical imaging by providing combined anatomical-metabolic image information. Especially in cancer patients, this may be of benefit.

PET itself is widely used in everyday routine due to its capability of providing molecular information used mainly for the non-invasive characterization of tumors and metastases [1,2], as well as for monitoring effect of cancer therapy [3]. However, PET images contain no detailed anatomical information and, therefore benefits from fusion with morphological image information from CT. Accordingly, since PET/CT systems became available oncological PET examinations have mainly been performed as combined PET/CT which has been proven of higher diagnostic value than separate PET or CT imaging in a number of clinical indications [4]. Recently, hybrid PET/MRI scanners were introduced and made available. However, PET/MRI is more costly and with a lower throughput than PET/CT. Accordingly, the question arises when and if PET/MRI should be used in cancer patients. MRI has the advantages compared to CT, that it may also be considered a functional imaging technique in addition to its anatomical capabilities. This may be of particular relevance in cancer patients in view of need for planning tailored therapy and for monitoring response to treatment [5,6]. Compared to CT, also the anatomical capabilities of MRI are often superior due to better soft-tissue contrast.

The knowledge obtained from molecular imaging-guided therapies e.g. using FDG-PET for predicting therapy response in lymphomas [7], could quickly be transferred to PET/MRI, for a combined multi-parametric strategy. Accordingly, hybrid PET/MRI scanners might become game-changers for how MRI is used in clinical routine.

At the Department of Clinical Physiology, Nuclear Medicine & PET at Rigshospitalet in Copenhagen we have currently PET/MRI scanned more than 1,200 patients and our experience so far will be presented and discussed with focus on whether PET/MRI fulfills a real clinical need within oncology or should (still) be considered an expensive research tool and the challenges of PET/MRI [8,9].
